# Reimagining the Terms Mongolian Spot and Sign

**DOI:** 10.7759/cureus.20396

**Published:** 2021-12-13

**Authors:** Steven Yale, Halil Tekiner, Eileen S Yale

**Affiliations:** 1 Internal Medicine, University of Central Florida College of Medicine, Orlando, USA; 2 Department of Medical History and Ethics, Erciyes University School of Medicine, Kayseri, TUR; 3 General Internal Medicine, University of Florida College of Medicine, Gainesville, USA

**Keywords:** slate grey nevus, blue-gray macules of infancy, ink-blot macule, congenital dermal melanocytosis, mongolian spot

## Abstract

The term “Mongolian Spot” rather than the preferred descriptive name congenital dermal melanocytosis (CDM) continues to be used despite compelling objections to the contrary. Terms that stigmatize a culture, region, people, country, communities, and ethnic group should be replaced by their more descriptive counterparts. Herein, we clarify terminology, discuss the historical significance, and provide a recommendation about naming this disease.

## Editorial

The term Mongolian Spot continues to be used within the most recent literature even though it is considered disparaging and marginalizing to a group of people. Our correspondence is intended to clarify terminology about this cutaneous physical finding and discuss the historical significance of the term Mongolian Spot in the context of more current guidelines and recommendations about naming diseases.

This cutaneous condition has been more appropriately termed and is now colloquially referred to as congenital dermal melanocytosis (CDM). Other alternative names proposed include ink-blot macules, blue-gray macules of infancy, or slate grey nevus [1,2]. The term CDM is a generic descriptive term that explains whom it affects (congenital), location (dermal), and cell type (melanocyte). A CDM represents a cutaneous disease detected by observation and is not a clinical sign. A sign like a physical finding is an objective marker but differs in representing an attribution, inference, or requires an interpretation of its significance. Thus, it provides meaning or an explanation to a finding or observation and thereby aid in identifying and diagnosing disease. The physical findings and features that assist in distinguishing a CDM from an ecchymosis are its stability, distribution, location, color, and being non-painful to touch.

Erwin Otto Eduard von Bälz (1849-1913) was credited for being the first to describe this clinical finding within the Japanese population in 1885 in his paper titled “Physical characteristics of the Japanese”:

Undisputedly, the most interesting pigmentation is a dark blue spot on the sacrum or buttock of all Japanese newborns. The stain is rarely found at other sites (e.g., one leg). This phenomenon first appears in the fifth fetal month (at the beginning of the second half of pregnancy) and soon disappears in the first two years of life only to become visible during childhood. I am unaware whether such a black and blue buttock is found elsewhere (e.g., in the children of parents with dark European complexion). I ignored this finding before, and books on the pigmentation of the skin have not discussed this topic. Nevertheless, it deserves the anatomist and physiologist’s attention, for, as far as I know, we have before us the only case in man in which pigment is found in the dermis (corium). The dye lies wholly or predominantly in the upper portion (epidermis) in all other physiological pigmentations. It appears here in its actual color as yellow and brown or in thicker layers as black, which is always the case when the dye sits in the faint, translucent portion of the dermis. How the stain arises is unclear; any particular intrauterine pressure cannot be implicated because the stain occurs evenly in cranial and buttock positions. The stain also appears much too early to account for such a development. A pressure mark would soon disappear after the birth. Finally, the microscopic findings are decisive; for a regularly occurring physiological appearance (p. 40) [3].

Bälz also identified the spot in the Mongolian people and erroneously believed that it represented a distinct characteristic finding within this race. Hence, he coined the German term “Mongolenfleck”. Our review of the PubMed/MEDLINE literature found that despite misconception and compelling evidence on the contrary regarding its anthropological lineage, misappropriation of the term to a specific race, and racial connotation, the term Mongolian Spot has unconventionally persisted within the medical literature from 1964 to 1993 and first reemerged after this hiatus in 2019. This finding illustrates how difficult it is to change a name once it becomes entrenched in the scientific taxonomical literature. Two recent papers published in 2019 by Zhong et al. and Prose called for exclusively adopting the term CDM [4,5]. We agree with this designation as it is a specific descriptive term (congenital) with histopathological relevance (dermal melanocytes).

Works by the National Institutes of Health and the World Health Organization (WHO) in classifying morphological or infectious diseases provide a theoretical framework that can be used for naming diseases in general. Although the WHO Best Practices refer to naming new infections, syndromes, and diseases, we believe certain principles are relevant and applicable to naming preexisting conditions including, Mongolian Spot, and should exclude those names that are non-benevolent and stigmatizing to a culture, regions, people, country, communities, and ethnic group. Thus, the name has an inappropriate and unpleasant connotation, and must be avoided.

## References

[1] Lin AE, Feingold M, Siegel B (1993). Out, out damn spot, or the demise of the Mongolian spot. Am J Dis Child.

[2] Kaplan RE (1984). Ink-blot macules: an alternative to “Mongolian spots”. Pediatr Dermatol.

[3] Bälz E (1885). Die körperlichen Eigenschaften der Japaner. Mitt dtsch Ges Natur-u Völkerkunde Ostasiens.

[4] Zhong CS, Huang JT, Nambudiri VE (2019). Revisiting the history of the "Mongolian spot": the background and implications of a medical term used today. Pediatr Dermatol.

[5] Prose NS (2019). Bringing an end to the "Mongolian Spot". Pediatr Dermatol.

